# Recovery of a Temperate Reef Assemblage in a Marine Protected Area following the Exclusion of Towed Demersal Fishing

**DOI:** 10.1371/journal.pone.0083883

**Published:** 2013-12-31

**Authors:** Emma V. Sheehan, Timothy F. Stevens, Sarah C. Gall, Sophie L. Cousens, Martin J. Attrill

**Affiliations:** 1 Marine Institute, Plymouth University, Drake Circus, Plymouth, United Kingdom; 2 Griffith School of Environment and Australian Rivers Institute - Coasts and Estuaries, Griffith University, Gold Coast, Queensland, Australia; The Australian National University, Australia

## Abstract

Marine Protected Areas MPA have been widely used over the last 2 decades to address human impacts on marine habitats within an ecosystem management context. Few studies have quantified recovery of temperate rocky reef communities following the cessation of scallop dredging or demersal trawling. This is critical information for the future management of these habitats to contribute towards conservation and fisheries targets.

The Lyme Bay MPA, in south west UK, has excluded towed demersal fishing gear from 206 km^2^ of sensitive reef habitat using a Statutory Instrument since July 2008.

To assess benthic recovery in this MPA we used a flying video array to survey macro epi-benthos annually from 2008 to 2011. 4 treatments (the New Closure, previously voluntarily Closed Controls and Near or Far Open to fishing Controls) were sampled to test a recovery hypothesis that was defined as ‘the New Closure becoming more similar to the Closed Controls and less similar to the Open Controls’.

Following the cessation of towed demersal fishing, within three years positive responses were observed for species richness, total abundance, assemblage composition and seven of 13 indicator taxa. Definitive evidence of recovery was noted for species richness and three of the indicator taxa (*Pentapora fascialis*, *Phallusia mammillata* and *Pecten maximus*).

While it is hoped that MPAs, which exclude anthropogenic disturbance, will allow functional restoration of goods and services provided by benthic communities, it is an unknown for temperate reef systems. Establishing the likely timescales for restoration is key to future marine management. We demonstrate the early stages of successful recruitment and link these to the potential wider ecosystem benefits including those to commercial fisheries.

## Introduction

Management of marine environments has historically been targeted towards maintaining commercial fish stocks, with conservation objectives coming second to economic imperatives [1]. Over the past two decades, studies have increasingly attempted to understand the wider effects of fishing and other human activities on the marine environment, resulting in a shift from fisheries-centred management to an ecosystem management approach [1], [2]. This type of management should not only benefit marine biodiversity, but should also feedback and benefit commercial fisheries by increasing the abundance of target species [3].

Marine Protected Areas (MPAs) can provide an effective ecosystem management approach to reducing the damaging effects of fishing on benthic assemblages and habitat [1], [4]–[9]. They can meet both fisheries management and conservation goals [1], [7] by protecting important and/or fragile habitat and preventing overfishing. Over time, well planned and managed MPAs can eventually enhance fisheries and facilitate the recovery of previously fished areas, known as spillover [10]–[12].

The performance of these MPAs must be assessed not only for management effectiveness, but also to ensure that governments comply with their management responsibilities. For example, EU countries are committed to establishing ecologically coherent networks of MPAs to enhance ecosystem health (Habitats Directive (92/43/EEC) and Birds Directive 79/409/EEC), and monitoring is therefore crucial to any assessment of their success. Establishing, enforcing and monitoring MPAs is costly and it is therefore also important to report their effectiveness to governments and to the public to encourage support for their use as marine conservation tools.

**Figure 1:**
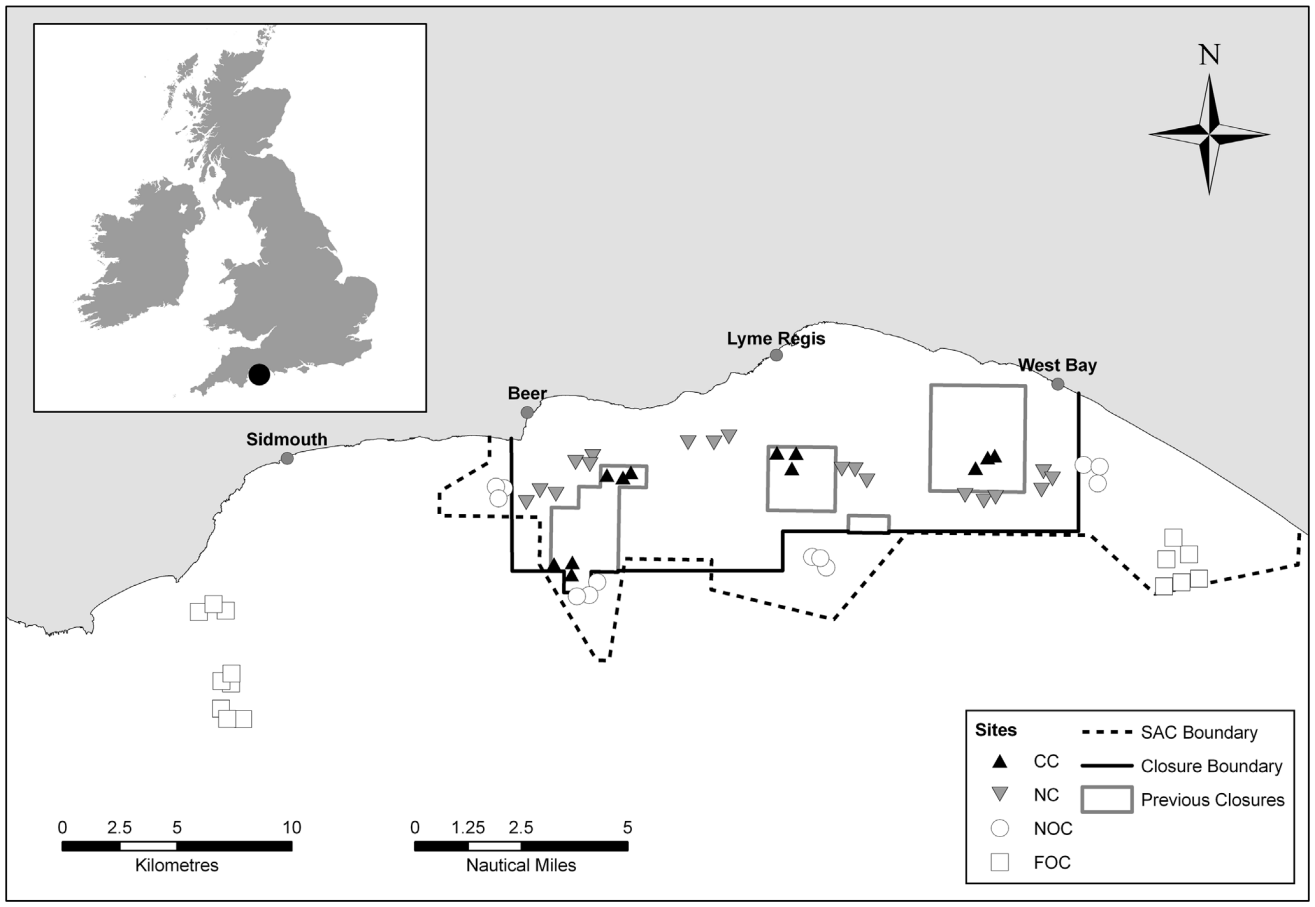
Locations of sites in Lyme Bay. Some symbols overlap at this scale.

Lyme Bay, on the south west coast of the UK (Fig. 1), is an area of high-biodiversity reefs formed of mudstone, limestone, chalk and granite outcrops, pebbles, cobbles and boulders, listed under Annex I of the Habitats Directive. These reefs are home to species including the iconic *Eunicella verrucosa* (Pallas, 1766) pink sea fan (listed under Schedule 5 of the UK Wildlife and Countryside Act 1981), the habitat-forming *Pentapora fascialis* (Pallas, 1766) ross coral and the commercially fished *Pecten maximus* (Linnaeus, 1758) [13] scallop. These constituent elements have allowed the site to be designated as an Annex 1 habitat ‘reefs’. Concerns have been raised over many years about the effects of towed demersal fishing gear, particularly scallop dredging that break up or overturn sections of fragile reef habitat and remove sessile fauna [3], [14], [15]. Many temperate reef sessile species are long lived and slow growing, and fishing disturbance is consequently long lasting and has been shown to have a substantial negative influence on benthic communities through changes in assemblage composition, trophic structure and habitat complexity [15]–[20].

Concerns raised over the impacts of towed demersal gear on Lyme Bay reef habitats were initially addressed through the creation of four small voluntary closures (totalling 22 km^2^), which were implemented in 2001 and 2006. Variable adherence to the voluntary agreements spurred continued support for one large MPA with greater levels of protection. In 2008, Lyme Bay became the UK's largest MPA under a Statutory Instrument (SI) protecting marine biodiversity through the exclusion of towed demersal fishing gear (scallop dredging and trawling) from a 206 km^2^ (60 nm^2^) area of seabed. Static gear fisheries, including potting and netting, were permitted to continue, along with diving for scallops and recreational activities, such as sea angling and SCUBA diving. The Lyme Bay closure is widely regarded as an important test site for UK and European marine conservation policy [21].

Effects of MPAs have been well reported for tropical systems [22], [23]. This is less well documented for temperate systems [19], [24]–[26], and this lack of information on the response of temperate reef fauna to protection meant that the recovery of Lyme Bay reef biodiversity was far from certain [27], [28]. Knowledge of recovery trends is, however, essential if MPAs are to be managed effectively to achieve conservation goals and be used as a tool to enhance fish stocks.

The aim of this study was to determine whether the biota of reef habitats within Lyme Bay showed evidence of recovery once the effects of scallop dredging and demersal trawling were removed (“passive recovery”, using the terminology of Elliott et al [29]).

## Materials and Methods

### Field surveys

Lyme Bay (Fig. 1) has a diverse range of benthic habitats, from rocky and cobble reefs to mixed pebbly sand and gravel sediments and muddy soft substrata. This study focused on those reefs defined by Annex I of the Habitats Directive as ‘habitats where animal and plant communities develop on rock or stable boulders and cobbles’ [30]. Annual surveys took place over the summer months from 2008–2011. The 2008 baseline survey took place six weeks after the implementation of the Statutory Instrument SI; however the anticipated changes in the benthic assemblage were expected to occur over annual or decadal time spans [31] so this was considered an adequate baseline. No specific permissions were required for these locations or activities as while some protected organisms were enumerated, no physical samples were taken, only video images. Field studies, therefore, did not involve sampling endangered or protected species across Lyme Bay (50° 34′ N, 3° 24′ W to 50° 37′ N, 2° 35′ W).

### Survey Design

The design of the study considered changes in abundance of epibenthic taxa annually from 2008 to 2011 within four treatment types. The “experimental” treatment was the new MPA, called ‘New Closure’ (NC), and this was compared to sites that continued to be fished; ‘Near Open Controls’ (NOC) within 5 km of the closure boundary, and ‘Far Open Controls’ (FOC) more than 5 km from the closure boundary. Sites were organised in Areas that were nested in Treatment. While there were no areas of Lyme Bay that could be considered “pristine”, the previously voluntarily protected areas had been nominally closed to dredging since either 2001 or 2006, so represented ‘Closed Controls’ (CC) for the purpose of this study. It is important to note that we do not assume that CC sites were completely unimpacted before the start of the study, but they represent areas of reef with the lowest past fishing activity (see site selection, below).

To assess recovery we tested the hypothesis that, subsequent to the closure of the Lyme Bay reefs to towed demersal fishing in 2008, the reef biota (measured as assemblage composition, species richness, total abundance, and abundance of pre-selected indicator taxa [32] in the NCs would increase relative to the open control sites (NOC, FOC) and would become more similar to the closed control sites (CC). Indicator taxa were selected based on life history, tolerance to disturbance and recoverability to represent the range of benthic fauna found in Lyme Bay. In addition to this narrow definition, and bearing in mind that the CC sites might benefit from the buffering effects of the statutory closure now surrounding them, we considered that increases in reef biota in both the NC and CC relative to the open control sites (NOC, FOC) would also constitute success of the MPA; we have, however kept these two scenarios separate in the results and discussion.

### Site Selection

To select candidate sites we conducted spatial analyses combining historical fishing effort, benthic substrate and biotope distribution, depth, and the boundaries of the SI and areas previously closed under voluntary agreements. Information on patterns of historical fishing effort was derived from vessel patrol sightings from 2005–2008 provided by Devon Sea Fisheries Committee (DSFC) and over-flight sightings from 2001–2007 provided by the Marine and Fisheries Agency (MFA). These data were used to construct a composite density plot of relative towed demersal fishing effort in five classes [21].

Data on benthic substrate and biotope distributions were provided by the Devon Biodiversity Records Centre, so that both reference and treatment sites could be located on similar substrates to avoid any habitat bias. Depth data was obtained from published admiralty charts. The boundaries of the SI and previous voluntary closures were added, since they in part define current patterns of use. These layers were merged to provide a single layer of polygons incorporating all the attributes of the source layers, enabling selection of those that met the necessary criteria. All sites were located on hard or “mixed” substrates (rock, boulders or cobbles). All sites were located between 15 and 25 m depth. Newly closed or open sites were located where scalloping effort was historically moderate to high, whereas closed sites were located where it was low (because they were within the voluntary closures) [21].

Final selection of four areas per treatment was conducted after ground-truthing at the commencement of the first sampling period; for example, local knowledge allowed the selection of sites of suitable habitat not identified in the existing habitat classification. Individual video frames (see below) were discarded if they were not located on rock or mixed boulders and cobble habitat. For this reason, while the target was to survey 3 sites for each Area, the number of sites suitable for analysis ranged from 2 to 5 sites per area. 60 useable video transects were analysed from 2008–2010, while 56 transects were analysed for 2011.

### Video surveys

A towed flying video array was developed to survey a 200 m×0.5 m video transect at each site in a non-destructive and cost-effective way [33]. In summary, the High Definition (HD) video system included a camera (Surveyor-HD-J12 colour zoom titanium, 720p), LED lights (Bowtech Products limited, LED-1600-13), two green laser pointers (Z-bolt Scuba-1) and a mini CTD profiler (Valeport Ltd). The umbilical was connected topside to a Bowtech System power supply/control unit allowing control of light intensity and camera focus, zoom and aperture. The camera was positioned at an oblique angle to the seabed, with the three lights fixed in front and below the camera to provide improved image definition and colour. The lasers were positioned parallel to each other at a known distance apart, so changes in the field of view with varying height above the substrate [34] could be quantified by measuring the apparent distance between the laser dots. This permits accurate determination of organism densities, without the need for a heavy and potentially damaging benthic sled [21].

### Video data extraction

Analysis of the video transects was conducted in two stages [33]. Firstly, infrequent/conspicuous fauna were counted from each entire video transect. Taxon counts were determined by viewing the video at normal speed, and recording each identifiable organism as it passed through the “gate” formed by the two laser dots. The position of the lasers in the field of view was noted during data extraction, and combined with the length of the tow from GPS positions, allowing the area surveyed to be calculated giving taxon abundance as density (individuals m^−2^). Secondly, frame grabs were extracted from the video at five second intervals (Cybertronix frame extractor) and a digital 0.25 m^2^ quadrat overlaid. Frame grabs were discarded if they were not in focus, overlapped each other, were not on the appropriate habitat or if the lasers were not within the acceptable margins of the quadrat overlay. Images would therefore only be selected if the camera was at an oblique angle to the seabed, which reduces potential error that may be introduced as a result of changing seabed slope. Analyses of a trial dataset comprising all possible frames from 12 video transects determined that using 30 frames gave equivalent result to extracting data from all frames, but with a substantial saving in processing time [21]. Individual or discrete colonial organisms counted within the 30 frames sub-sampled from each video transect were expressed as densities (individuals m^−2^). The quadrat overlay contained 16 dots. Cover-forming colonial taxa were quantified as percent cover by dividing the number of dots overlying that taxon by the total number of dots for the quadrat.

All organisms present were identified to the highest taxonomic level possible and their abundance recorded. Taxonomically similar species, which could not be distinguished with confidence, were grouped. Such groups included: *Inachus* spp. and *Macropodia* spp. (identified to genus level); Gobies; Hydroids (excepting *Nemertesia antennina* (Linnaeus, 1758), *Gymnangium montagui* (Billard, 1912) and *Nemertesia ramosa* (*Lamouroux, 1816*)) and Branching sponges. The category Turf incorporated hydroids and bryozoans that were <1 cm.

### Data analyses

Permutational Multivariate Analysis of Variance (PERMANOVA+, in the PRIMER v6 software package [35], [36]) was used to test for changes in the response variables (species richness, total abundance, assemblage composition and the abundance of pre-selected indicator taxa [32]) in the NC relative to the CC, NOC and FOC, over temporal and spatial scales. Analyses of species richness, total abundance and assemblage composition used frame grab data. For analyses of the 13 indicator taxa, five taxa used frame grab data, while the remainder used data from the entire video transect. PERMANOVA is robust to datasets with many zeros, and allows the testing of interactions in complex multifactorial designs with multivariate or univariate data. It has significant advantages over conventional MANOVA in that it makes no assumptions about underlying data distributions, and is robust to unbalanced designs [37].

Multivariate data (assemblage) were dispersion weighted and square root transformed. Bray-Curtis similarity indices were calculated from Sites × Taxa abundance data to construct a similarity matrix between sites [38]. Dispersion weighting was employed to down-weight taxa with large and erratic numbers without ‘squashing’ other taxa [39] and a square root transformation was then applied to allow the rare taxa to contribute to the outcome, and further down-weight high-abundance taxa. Visualisation of the dissimilarity matrices was achieved using non-metric Multi-Dimensional Scaling (nMDS). Univariate data (species richness, total abundance and indicator taxa) were log_10_(x+1) transformed and Euclidean distance indices were used to construct similarity matrices between sites [40].

The analytical design had four factors: Year (fixed: 2008, 2009, 2010, 2011), Treatment (fixed: CC, NC, NOC, FOC), Area (random and nested in Treatment), and Site (random and nested in Treatment and Area). Within-transect variation was not of interest given the scale of the study, so the 30 replicate frame grabs were averaged to avoid pseudoreplication. This also increased the precision at which the epibenthic assemblage was quantified.

Each term in the analyses used 9999 permutations of the appropriate units [38]. Multi-level significant interactions were tested using PERMANOVA pairwise tests.

## Results

A total of 136 taxa from 9 phyla were recorded in the surveys: 125 taxa in the frame grab analysis and 46 in the video analysis. While frame grabs were only analysed if they were on ‘reef’ habitat (which constituted seabed with rock, boulders and cobbles), reef associated fauna, such as soft corals *Alcyonium digitatum* (Linnaeus, 1758) and upright bryozoans *Pentapora fascialis*, were also observed on sediments that appeared to overlay bedrock [41].

### Species richness

**Figure 2:**
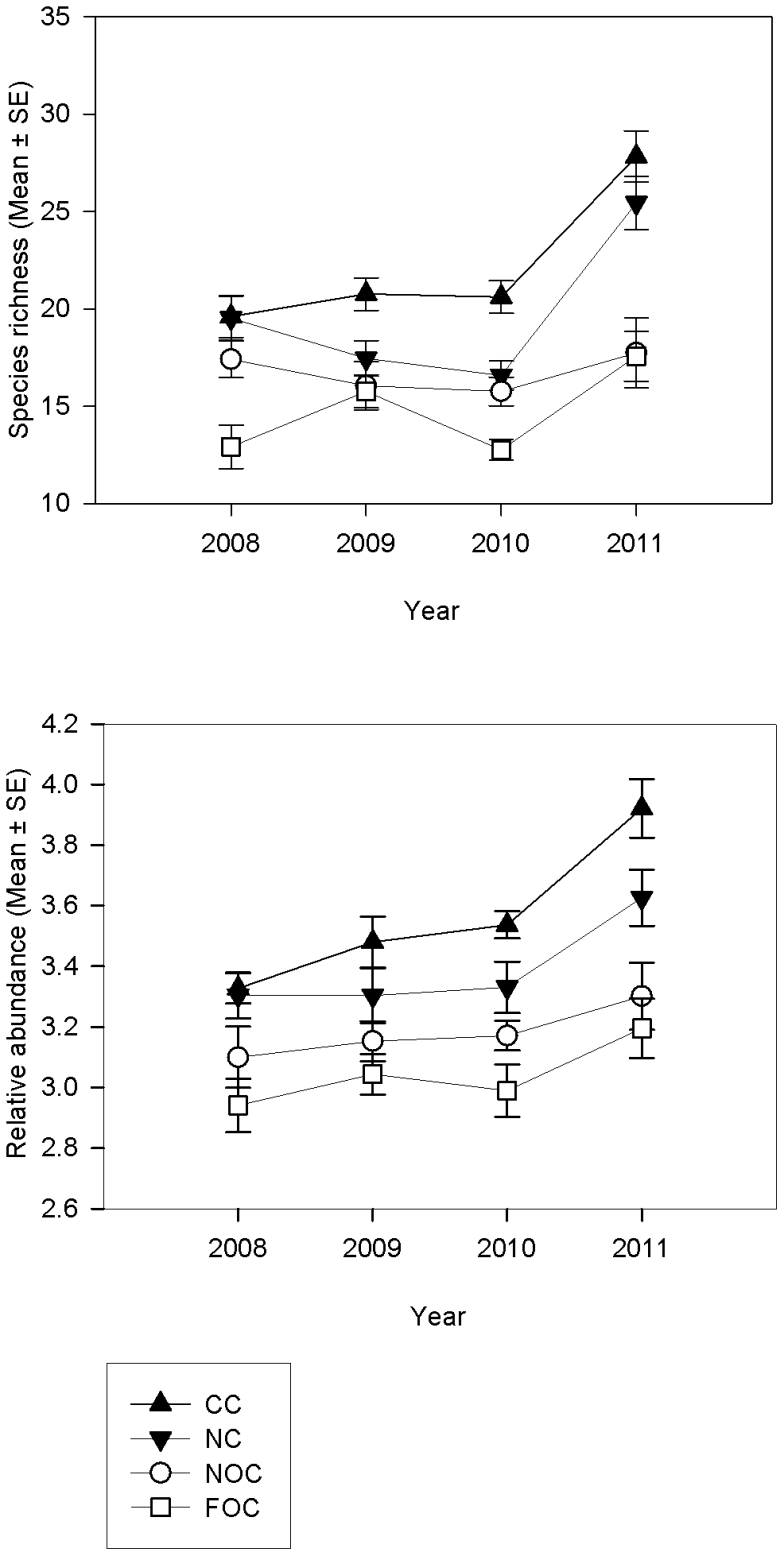
Univariate diversity measures to assess benthic recovery a) Species richness (mean m^−2^ ± SE) and b) Total abundance of all taxa within frame grabs, (mean m^−2^ ± SE), over time (2008, 2009, 2010, 2011) and treatment type (CC  =  closed control, NC  =  new closure, NOC  =  near open control, FOC  =  far open control).

Species richness was greatest in the CC in 2011 (27.8 m^−2^±1.32) and lowest in the FOC in 2010 (12.77 m^−2^±0.53) (Fig. 2a; Table 1). A significant Year × Treatment interaction indicated that species richness differences between treatments varied over time. Clear trends were not apparent for the first two years of the study, but by 2011 the species richness in the NC (25.44 m^−2^±1.37) was greater than in both the NOC and FOC (NOC: 17.75 m^−2^±1.8; FOC: 17.57 m^−2^±1.28) and was not different to the CC (27.83 m^−2^±1.32). Significant variation was identified between sites nested within area (*P* = 0.012), demonstrating the high degree of small scale spatial variation across the study site (Table 1). Perhaps surprisingly, for both species richness and total abundance (below), NC and CC values were very similar at the outset (2008), and diverged thereafter, although they both diverged further from the open control sites.

**Table 1:** PERMANOVA of species richness based on Euclidean distance measure.

a)								
Source	*df*	SS	MS	*F*	P			
Year Ye	3	1213.30	404.43	20.52	**0.0001**			
Treatment Tr	3	1697.20	565.74	10.61	**0.0002**			
Area Ar (Tr)	15	713.08	47.539	2.04	**0.0358**			
YexTr	9	410.27	45.585	2.55	**0.0171**			
Site(Ar(Tr))	50	1061.30	21.225	1.72	**0.0117**			
YexAr(Tr)	45	690.03	15.334	1.25	0.1819			
Residual	110	1354.70	12.315					
Total	235	7139.80						

b)								
	2008		2009		2010		2011	
Groups	t	P	t	P	T	P	t	P
CC, NC	0.73	0.5506	1.81	0.093	2.57	**0.0233**	0.94	0.3938
CC, NOC	1.71	0.1275	2.33	0.0541	3.47	**0.0091**	4.66	**0.0274**
CC, FOC	4.04	**0.0032**	2.46	**0.0382**	5.08	**0.001**	4.14	**0.0043**
NC, NOC	2.57	**0.0229**	0.97	0.3862	0.86	0.4705	2.73	**0.0271**
NC, FOC	5.58	**0.0001**	1.08	0.3225	3.09	**0.0088**	2.79	**0.0216**
NOC, FOC	3.12	**0.0134**	0.40	0.8904	3.08	**0.0149**	0.11	0.9891

a) Main test and b) Pairwise testing for the interaction Year × Treatment. Data were Log (x+1) transformed. Bold type denotes a significant result.

### Total abundance

Total abundance calculated from the frame grabs was greatest in the CC in 2011 (3.9 m^−2^±0.1) and lowest in the FOC in 2008 (2.94 m^−2^±0.09), (Fig. 2b). Abundance differed between treatments and years (*P*<0.05) and was significantly greater in the CC (3.57 m^−2^±0.07) than the NOC (3.18 m^−2^±0.0.08) or FOC (3.04 m^−2^±0.08). Abundance in the NC (3.39 m^−2^±0.08) was also greater than the FOC, and was greater in 2011 (3.51 m^−2^±0.1) than any other year (2008 = 3.17 m^−2^±0.08; 2009 = 3.25 m^−2^±0.08; 2010 = 3.26 m^−2^±0.07; all *P*<0.001; Fig. 2b; Table 2). While there appears to be increased abundance in the NC and CC relative to the fished treatments (Fig. 2b), there was no Year × Treatment interaction and so differences were not yet a significant indication of recovery as defined.

**Table 2:** PERMANOVA of abundance based on Euclidean distance measure.

a)					
Source	*df*	SS	MS	*F*	P
Year Ye	3	3.79	1.26	12.97	**0.0001**
Treatment Tr	3	8.44	2.81	5.13	**0.01**
Area Ar (Tr)	15	7.35	0.49	4.60	**0.0001**
YexTr	9	0.53	0.06	0.81	0.6059
Site(Ar(Tr))	50	4.75	0.10	1.45	0.0626
YexAr(Tr)	45	3.34	0.07	1.13	0.2934
Residual	110	7.19	0.07		
Total	235	35.39			

b)					
	Tr			Ye	
Groups	t	P	Groups	t	P
CC, NC	1.23	0.2574	2008, 2009	1.08	0.2928
CC, NOC	3.75	**0.0091**	2008, 2010	1.87	0.0793
CC, FOC	3.52	**0.009**	2008, 2011	4.77	**0.0001**
NC, NOC	1.61	0.1326	2009, 2010	0.45	0.6634
NC, FOC	2.30	**0.0473**	2009, 2011	4.35	**0.0002**
NOC, FOC	1.13	0.3055	2010, 2011	4.40	**0.0005**

a) Main test and b) Pairwise testing for the interactions Treatment and Year. Data were Log (x+1) transformed. Bold type denotes a significant result.

### Assemblage composition

**Figure 3:**
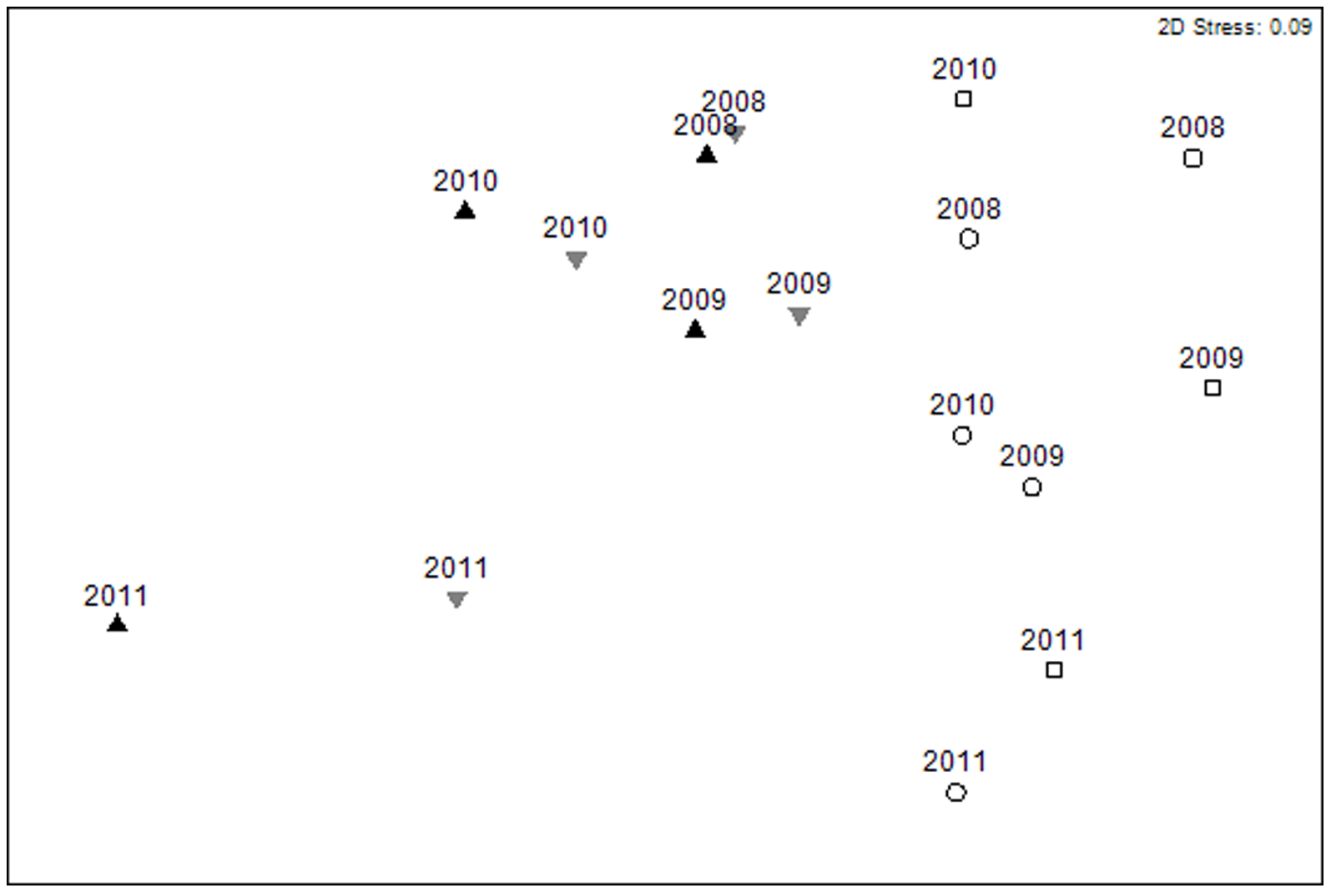
nMDS plot illustrating similarities in assemblage composition between Treatments (averaged for site within treatment), (closed control  =  filled black triangles, new closure  =  filled grey triangles, near open control  =  open circle, far open control  =  open square), over time (2008, 2009, 2010 and 2011). Data were dispersion weighted and square root transformed. Trajectories over time are indicated with lines from 2008 to 2011 for each treatment.

Assemblage composition was significantly different for every factor tested (Table 3a). Pairwise tests for Year × Treatment interaction showed significant differences for all years between the NC and FOC and the CC and FOC (all *P*<0.01, Table 3b). In 2008, the assemblages in the NC and NOC and CC were not different but became significantly different between protected and fished treatments by 2009. These differences remained consistent into 2010 and 2011. However, the nMDS (Fig. 3) showed that the while assemblage composition in the NCs continued to diverge from the open controls and shift toward the CCs, the CCs themselves diverged even further from the open controls, with the result that the NCs also became less similar to the CCs over time (Fig. 3).

**Table 3:** PERMANOVA of assemblage composition based on Bray Curtis similarity measure.

a)								
Source	*df*	SS	MS	*F*	P			
Year Ye	3	45921	15307	7.40	**0.0001**			
Treatment Tr	3	48855	16285	3.31	**0.0006**			
Area Ar (Tr)	15	66234	4415.6	3.36	**0.0001**			
YexTr	9	24506	2722.9	1.45	**0.0055**			
Site(Ar(Tr))	50	59146	1182.9	1.46	**0.0001**			
YexAr(Tr)	45	73742	1638.7	2.03	**0.0001**			
Residual	110	88847	807.7					
Total	235	407250						

b)								
	2008		2009		2010		2011	
Groups	t	P	t	P	t	P	t	P
CC, NC	0.77	0.91	0.86	0.7216	1.04	0.3788	1.33	0.0959
CC, NOC	1.03	0.3914	1.59	**0.0384**	1.85	**0.01**	2.46	**0.0269**
CC, FOC	1.57	**0.029**	1.70	**0.0266**	1.72	**0.021**	2.36	**0.0077**
NC, NOC	0.99	0.4642	1.59	**0.02**	1.67	**0.0069**	1.95	**0.0299**
NC, FOC	1.70	**0.0056**	1.60	**0.0268**	1.50	**0.0348**	2.02	**0.0138**
NOC, FOC	1.17	0.1774	1.43	0.0565	1.18	0.1606	1.01	0.4466

a) Main test and b) Pairwise testing for the interaction Year × Treatment. Data were dispersion weighted and square root transformed. Bold type denotes a significant result.

### Indicator taxa

#### Sessile indicator taxa

**Figure 4:**
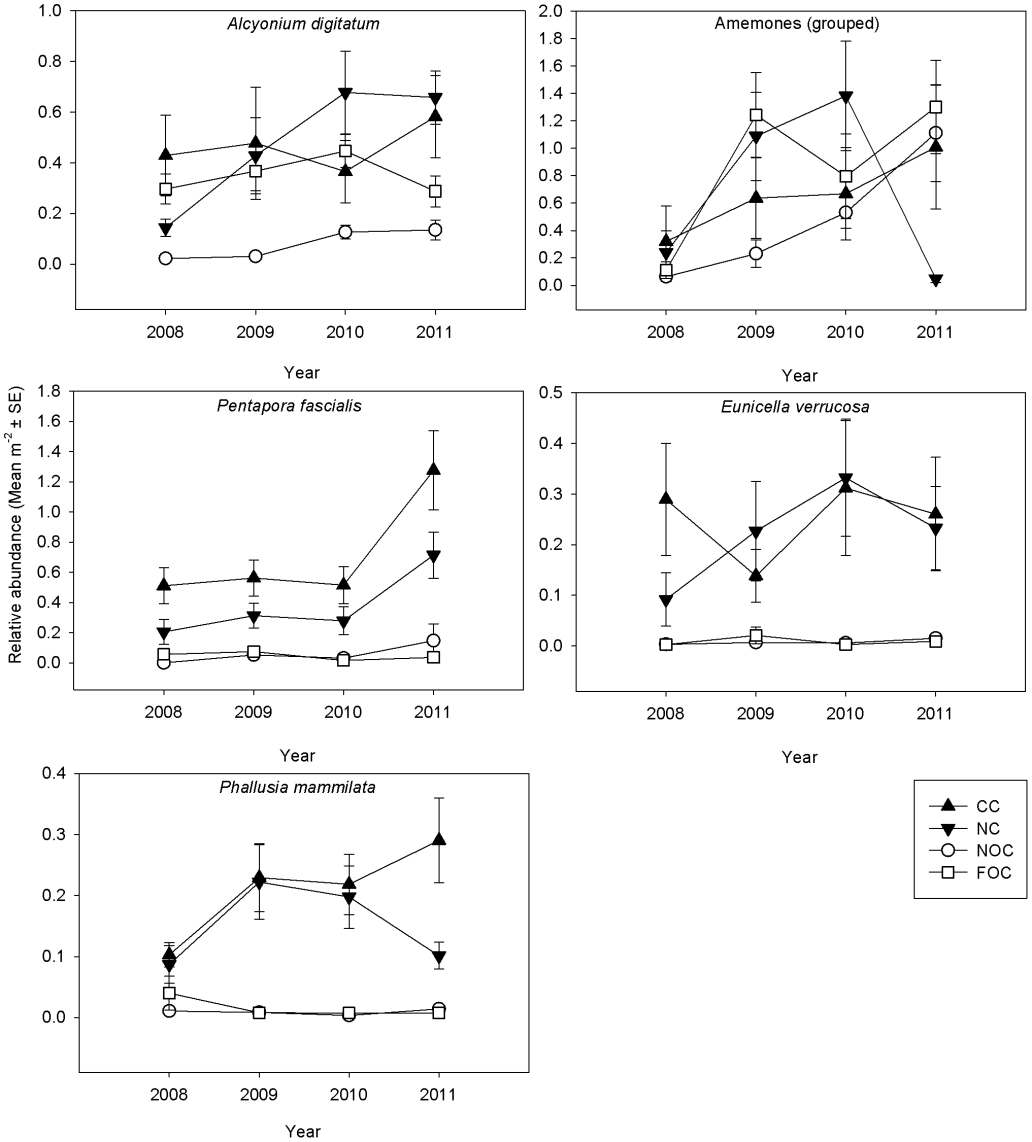
Relative abundance of sessile indicator species (mean m^−2^ ± SE) per treatment (CC  =  closed control (black triangle), NC  =  new closure (grey triangle), NOC  =  near open control (white circle), FOC  =  far open control (white square), per year (2008, 2009, 2010, 2011) identified through frame grabs.

**Figure 5:**
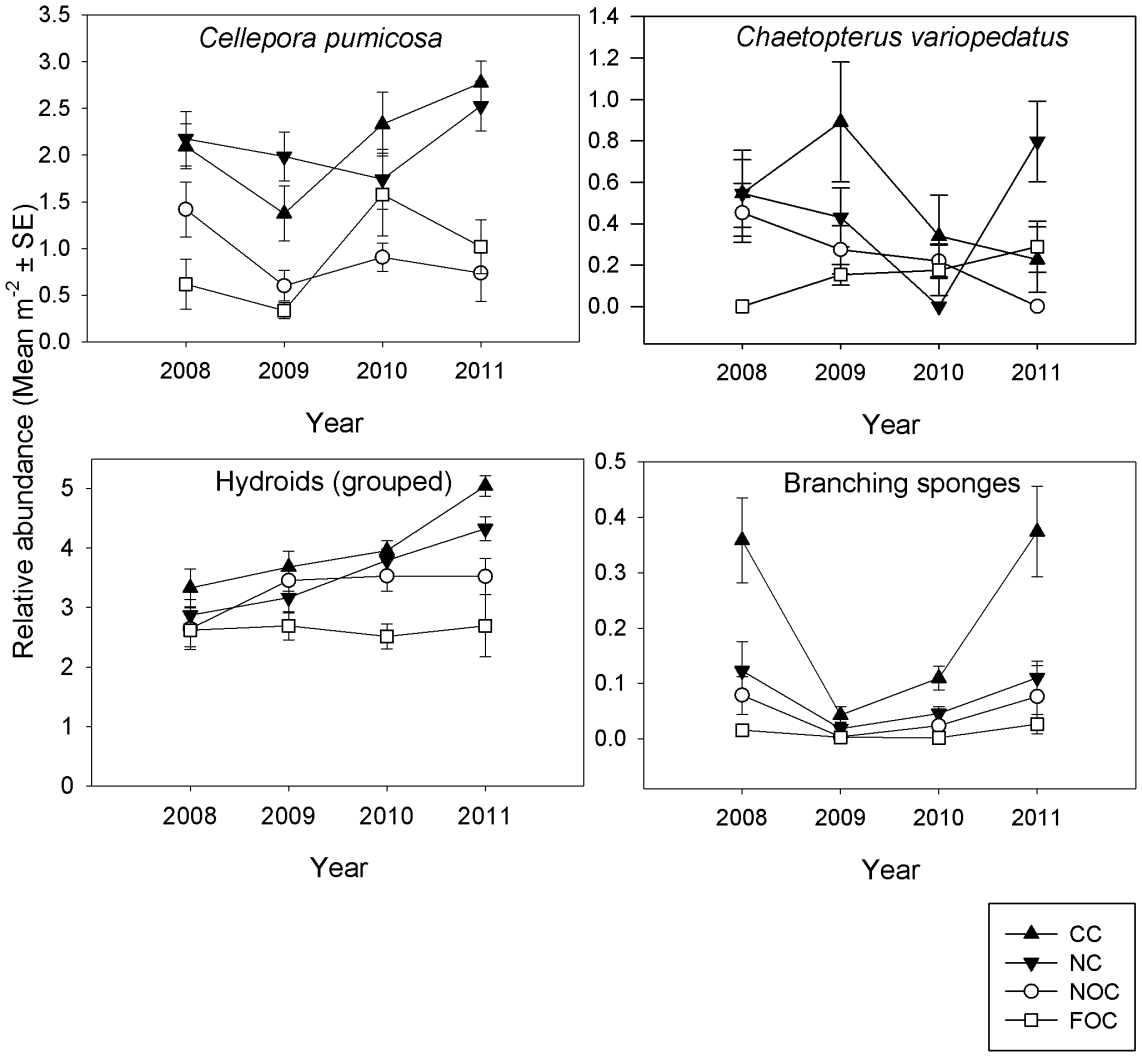
Relative abundance of sessile indicator species (mean m^−2^ ± SE) per treatment (CC  =  closed control, NC  =  new closure, NOC  =  near open control, FOC  =  far open control), per year (2008, 2009, 2010, 2011) identified through video transects.

Despite marked spatial variation across the bay, there was clear evidence of recovery for two of the nine sessile indicator taxa (*P. fascialis* and *Phallusia mammillata* (Cuvier, 1815), (Fig. 4, Table 4), and evidence of a positive response in a further three taxa (*A. digitatum*, *E. verrucosa* amd Grouped Hydroids; Figs. 4 and 5, Table 4). The spatial variation detected within treatment for the random area and site factors will not be further interpreted as hypotheses were specific to relative change in treatment over time. Overall *A. digitatum* dead man's fingers and Grouped Anemones were significantly more abundant in 2011 than 2008, but there was substantial spatial variation unrelated to treatment (Fig.4, Tables S1 and S2). Signs of recovery for *P. fascialis* ross coral were indicated by a significant Year × Treatment interaction (*P*<0.05) (Fig. 4; Table S3), and over time, abundance increased in both protected treatments. By 2011, there was a greater abundance in the CC than in the NC and similarly more in the NC than the fished treatments (Table S3).

**Table 4:** Summary of recovery status with evidence from pairwise statistical tests (in supporting information).

Response metric	Data Type	Recovery	Positive response
Species richness	Frames	Yes	Yes
Total Abundance	Frames	No	Yes
Assemblage composition	Frames	No	Yes
Sessile indicator taxa			
Branching sponges	Video	No	No
*Phallusia mammillata*	Video	Yes	Yes
*Alcyonium digitatum*	Video	No	Yes
*Eunicella verrucosa*	Video	No	Yes
*Chaetopterus variopedatus*	Frames	No	No
Hydroids (grouped)	Frames	No	Yes
*Cellepora pumicosa*	Frames	No	No
*Pentapora fascialis*	Frames	Yes	Yes
Anemones (grouped)	Frames	No	No
Mobile indicator taxa			
*Asterias rubens*	Video	No	No
*Necora puber*	Video	No	Yes
*Cancer pagurus*	Video	No	No
*Pecten maximus*	Video	Yes	Yes

*Data Type* refers to data quantified from the 30-frame subsample (*Frames*) or counts over the entire video transect (*Video*). *Recovery* is used in the narrow sense where NC increases relative to NOC & FOC, and approaches CC. *Positive response* indicates that NC increases relative to NOC & FOC, but does not necessarily converge with CC, in that CC may also increase, or show wide variability.

Substantial spatial variation was detected for the abundance of *E. verrucosa* pink sea fan. The trend shows a marked increase in *E. verrucosa* in the non-fished treatments compared to those that continued to be fished between 2010 and 2011 (Fig. 4) but there was no Year × Treatment interaction to determine a formal recovery trend as defined (Table S4).

While the null hypothesis of no recovery cannot be rejected, there was a strong signal (*P* = 0.53; Table S5) and trend (Fig. 4) that populations of *P. mammillata* which were distributed evenly across treatments in 2008 in the bay were increasing in the NC and CC relative to fished controls.

The abundance of *C. pumicosa*, a small, relatively tough bryozoan, differed significantly with Treatment and Area nested within Treatment (*P*<0.05; Table S6), which could be attributed to spatial differences rather than those associated with the closure (Fig. 5). The overall trend suggests that *C. pumicosa* is increasing in the protected treatments relative to the controls, but there is substantial variability in this population.

A Year × Treatment interaction of the abundance of *Chaetopterus variopedatus* (Renier, 1804) parchment worm indicated a difference between treatments over time (Fig. 5; Table S7). By 2011 the abundance of the polychaete was significantly greater in the NC than the NOC sites (*P*<0.05). Generally, however, the pairwise tests did not show a clear recovery trend.

There were significantly more Grouped Hydroids in 2011 than in 2008 (*P* = 0.0006) but treatment differences did not vary over time (Table S8). The graph shows an increasing abundance of Hydroids in the protected treatments but there was great spatial variation, which makes any recovery trends difficult to detect at present (Fig. 5).

The abundance of Branching Sponges varied over years and treatment (Fig. 5; Table S9) and appeared to show relative positive change in the CC compared to all other treatments, but there was no Year × Treatment interaction.

#### Mobile indicator taxa

**Figure 6:**
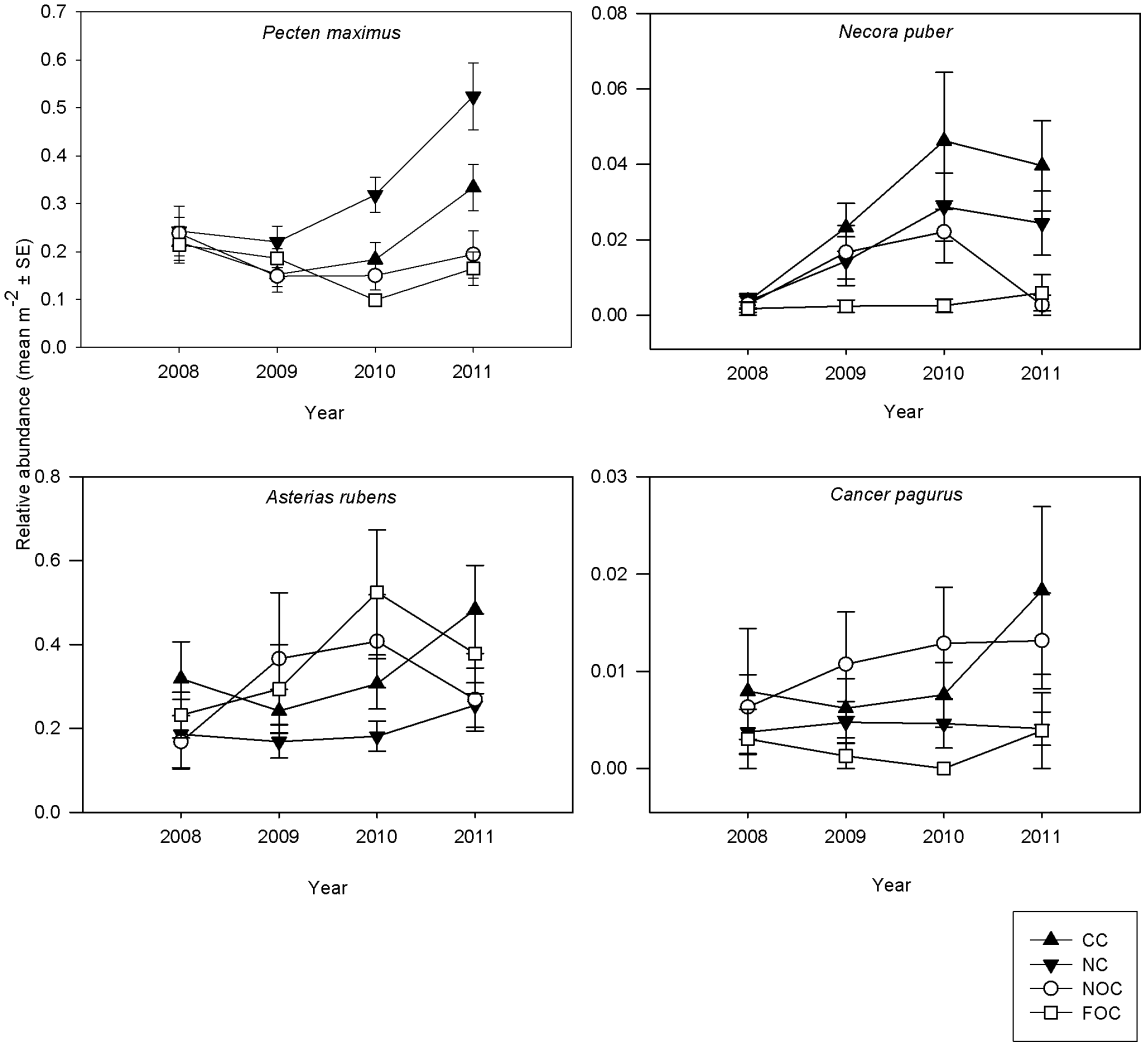
Relative abundance (mean m^−2^ ± SE) of mobile indicator species per treatment (CC  =  closed control, NC  =  new closure, NOC  =  near open control, FOC  =  far open control), per year (2008, 2009, 2010, 2011).

Significant evidence of recovery was apparent for one of the four mobile indicator taxa (*P. maximus*; Fig. 6, Table 4), and evidence of a positive response for another two (*A. rubens* and *Necora puber*) (Fig. 6, Table 4). The great scallop *P. maximus*, one of the main commercial target species in Lyme Bay, was observed in similar abundances across the treatments at the time of the baseline survey in 2008. By 2010, however, there were more *P. maximus* in the NC than in both of the open controls NOCs and FOCs (Ye × Tr interaction *P*<0.05, followed by pairwise tests; Fig. 6; Table S10).

There were significantly more velvet swimming crabs *N. puber* in 2011 than in 2008 (*P* = 0.0175), and evidence of an increasing trend in closed areas, but there was great spatial variation (Fig. 6; Table S11).

The abundance of the common sea star *A. rubens* was found to differ significantly between Area nested within Treatment (*P* = 0.0001), but no treatment or year effects were found (Fig. 6; Table S12).

A significant treatment effect was found for the edible crab *Cancer pagurus* (*P*<0.05) whose abundance was found to differ significantly, with more crabs in the NOC than the FOC sites (*P*<0.05) (Fig. 6; Table S13).

## Discussion

In 2008, when the MPA in Lyme Bay was designated and the first survey was undertaken, boulders and cobbles inside the newly closed areas had limited sessile life growing on them. This was most likely a result of the scraping action of destructive fishing gear that overturns boulders thereby crushing or removing the attached sessile, slow growing organisms. Three years later, there were significant relative changes indicating some recovery of the epi-benthic fauna.

Our definition for “recovery” required sites within the new closure (NC) to become more similar to those within the closed controls (CC) and less similar those that remained open to towed demersal fishing (NOC, FOC). We also considered a less stringent test, in the context of possible buffering of the CC sites by the NC which now surrounds them, characterized as a “positive response” where both the CC and the NC sites increase (or change, in the case of assemblage composition) relative to controls. Changes in the metrics measured in each treatment were used to determine whether this hypothesis could be accepted. These showed that species richness in the NC became significantly greater within 3 years of protection than that in the NOC and FOC; the abundance of fauna increased over the 3 years, but did not change significantly within the NC compared to the NOC and FOC; and the species assemblage in the NC became less similar to the fished treatments, but also less similar over time to the CCs.

Taken broadly, we conclude that positive changes were occurring within the NC, and that CC sites were also changing, perhaps benefitting from the buffering effect of the NC and the added protection offered by the SI rather than the previous voluntary agreements [42], [43]. This meets our definition of positive response but not recovery at this time. It is expected that the assemblage structure in the CC and the NC will eventually converge and remain dissimilar to the NOC and FOC allowing the formal recovery hypothesis to be accepted. Determining how long this will take is very important for marine ecosystem management. Evidence suggests decadal timespans may be required [44].

Within the first three years of the MPA three out of the 13 indicator species (*Pentapora fascialis*, *Phallusia mammilata* and *Pecten maximus*) showed recovery in the new closure. This is particularly of note for *P. fascialis*, a species that was previously known to be impacted by scallop dredging [20], with apparent low recoverability, as it is a functionally important bioconstructor which plays a key role in the formation of biogenic reef [45], [46]. Such species are known to improve survivorship of taxa such as juvenile fish through the provision of a structurally complex habitat [47], so its increased abundance is particularly encouraging for the recovery of closed sites. By 2011, *P. fascialis* presence will therefore help to create important fishery nursery areas and feeding grounds [26], [47]–[49].

A further five taxa showed a positive response: Grouped Hydroids, *Alcyonium digitatum*, *Eunicella verrucosa, Asterias rubens* (Linnaeus, 1758) and *Necora puber* (Linnaeus, 1767). There was considerable variation across the study area, but with time the early trends apparent for these or other taxa may consolidate the recovery picture.

The enhanced structural complexity of biogenic reefs, including hydroids, bryozoans and seafans, slows water movement and helps stabilise sediments [47]. Increased structural complexity supports both greater productivity and biodiversity by increasing the surface area and the range of habitat types available for settlement [47]. In turn, as assemblage diversity increases so does resilience to future impacts (including climate change) because of redundancy in trophic structure. More productive assemblages capture and recycle water column nutrients through filter feeding [50], and produce planktonic larvae that supports higher trophic levels. This bentho-pelagic coupling through a range of trophic links provides prey for birds [51], and commercially important fishes such as cod (*Gadus morhua*) [52].

It is important to note that the main target species of the excluded fishery, the commercially valuable great scallop *P. maximus* (DEFRA, 2012) was also found to be in a state of recovery inside the MPA despite a previous study concluding that scallops were not affected by bottom fishing in Lyme Bay [20]. Survey work by Hinz et al [20], which took place a year before the statutory instrument was introduced found no difference of *P. maximus* abundance between fished and non-fished treatments. The present study also found no difference in *P. maximus* abundance between all four treatments in 2008, but by 2011 abundance was significantly greater in the new closure than all other treatments. This suggests that *P. maximus* was impacted across the bay before the statutory instrument was in place but Hinz et al [20] were unable to detect this due to a lack of suitable controls. A similar study [53], assessing the north-east American *Placopecten magellanicus* population, identified a greater abundance of scallops within areas closed to mobile fishing gear. It would be expected that, with time, the protection of the SI will, in the long term, increase the survival of *P. maximus*, leading to a more stable and fecund population as large individuals become more abundant [19], [54]. This could result in spillover of individuals from the SI into the fished areas, benefitting the scallop dredge fishery in the bay. Variable results for the abundance of the edible crab *Cancer pagurus* (Linnaeus, 1758) suggest early evidence of spillover as abundance in the NOC increased in 2010 and was greater than in the closed treatments where intensive potting continued. This was also in stark contrast to abundances within the FOC, suggesting that crabs could be moving out to habitats close to the edge of the SI from within the MPA.

In summary, the results after three years of protection are broadly consistent with the international experience. A range of MPA-related studies have reported detectable trends towards recovery within the space of a few years e.g. [54]–[56], but in many cases more complete recovery occurs at decadal time-scales e.g. [44]. It is, therefore, critical that the closure remains in place while the long term study continues, to determine the time spans of recovery for benthic assemblages.

## Supporting Information

Table S1 **PERMANOVA of** ***Alcyonium digitatum*** **abundance based on Bray Curtis similarity measure and b) Pairwise testing for the interaction Ye.** Data were dispersion weighted and square root transformed. Bold type denotes a significant result.
(DOCX)

Table S2 **PERMANOVA of anemone abundance based on Bray Curtis similarity measure and b) Pairwise testing for the interaction Ye.** Data were dispersion weighted and square root transformed. Bold type denotes a significant result.
(DOCX)

Table S3 **PERMANOVA of** ***Pentapora fascialis*** **abundance based on Bray Curtis similarity measure and b) Pairwise testing for the interaction YexTr.** Data were dispersion weighted and square root transformed. Bold type denotes a significant result.
(DOCX)

Table S4 **PERMANOVA of** ***Eunicella verrucosa*** **abundance based on Bray Curtis similarity measure.** Data were dispersion weighted and square root transformed. Bold type denotes a significant result.
(DOCX)

Table S5 **PERMANOVA of** ***Phallusia mammillata*** **abundance based on Bray Curtis similarity measure.** Data were dispersion weighted and square root transformed. Bold type denotes a significant result.
(DOCX)

Table S6 **PERMANOVA of** ***Cellepora pumicosa*** **abundance based on Bray Curtis similarity measure.** Data were dispersion weighted and square root transformed. Bold type denotes a significant result.
(DOCX)

Table S7 **PERMANOVA of** ***Chaetopterus variopedatus*** **abundance based on Bray Curtis similarity measure and b) Pairwise testing for the interaction YexTr.** Data were dispersion weighted and square root transformed. Bold type denotes a significant result.
(DOCX)

Table S8 **PERMANOVA of hydroid abundance based on Bray Curtis similarity measure and b) Pairwise testing for the interactions Tr and Ye.** Data were dispersion weighted and square root transformed. Bold type denotes a significant result.
(DOCX)

Table S9 **PERMANOVA of branching sponge abundance based on Bray Curtis similarity measure and b) Pairwise testing for the interaction YexTr.** Data were dispersion weighted and square root transformed. Bold type denotes a significant result.
(DOCX)

Table S10 **PERMANOVA of** ***Pecten maximus*** **abundance based on Bray Curtis similarity measure and b) Pairwise testing for the interaction YexTr.** Data were dispersion weighted and square root transformed. Bold type denotes a significant result.
(DOCX)

Table S11 **PERMANOVA of** ***Necora puber*** **abundance based on Bray Curtis similarity measure and b) Pairwise testing for the interaction Ye.** Data were dispersion weighted and square root transformed. Bold type denotes a significant result.
(DOCX)

Table S12 **PERMANOVA of** ***Asterias rubens*** **abundance based on Bray Curtis similarity measure.** Data were dispersion weighted and square root transformed. Bold type denotes a significant result.
(DOCX)

Table S13 **PERMANOVA of** ***Cancer pagurus*** **abundance based on Bray Curtis similarity measure and b) Pairwise testing for the interaction Tr.** Data were dispersion weighted and square root transformed. Bold type denotes a significant result.
(DOCX)
