# Supplementary material for: Recovery of a Temperate Reef Assemblage in a Marine Protected Area following the Exclusion of Towed Demersal Fishing
Source: PLoS One. 2013 Dec 31;8(12):e83883. doi: 10.1371/journal.pone.0083883 (PMC3877100; doi:10.1371/journal.pone.0083883)
Supplement: Table S1 — PERMANOVA of Alcyonium digitatum abundance based on Bray Curtis similarity measure and b) Pairwise testing for the interaction Ye. Data were dispersion weighted and square root transformed. Bold type denotes a significant result. (DOCX) [file pone.0083883.s001.docx]

Table S1: PERMANOVA of *Alcyonium digitatum* abundance based on Bray Curtis similarity measure and b) Pairwise testing for the interaction Ye. Data were dispersion weighted and square root transformed. Bold type denotes a significant result.

| **a)** |  |  |  |  |  |
| --- | --- | --- | --- | --- | --- |
| **Source** | **df** | **SS** | **MS** | **F** | **P** |
| Year Ye | 3 | 1.81 | 0.60166 | 2.79 | **0.0383** |
| Treatment Tr | 3 | 6.69 | 2.2307 | 0.92 | 0.4617 |
| Area Ar (Tr) | 15 | 32.21 | 2.1476 | 22.61 | **0.0001** |
| YexTr | 9 | 2.06 | 0.22884 | 1.29 | 0.2629 |
| Site(Ar(Tr)) | 59 | 4.80 | 0.081329 | 2.14 | **0.0007** |
| YexAr(Tr) | 45 | 6.60 | 0.14661 | 3.86 | **0.0001** |
| Residual | 117 | 4.45 | 0.03801 |  |  |
| Total | 251 | 58.61 |  |  |  |

| **b)** |  | |
| --- | --- | --- |
|  | **Ye** | |
| **Groups** | **t** | **P** |
| 2008, 2009 | 1.20 | 0.2578 |
| 2008, 2010 | 2.00 | 0.0505 |
| 2008, 2011 | 3.00 | **0.006** |
| 2009, 2010 | 1.14 | 0.2788 |
| 2009, 2011 | 1.31 | 0.2039 |
| 2010, 2011 | 0.53 | 0.6042 |
