# Supplementary material for: Recovery of a Temperate Reef Assemblage in a Marine Protected Area following the Exclusion of Towed Demersal Fishing
Source: PLoS One. 2013 Dec 31;8(12):e83883. doi: 10.1371/journal.pone.0083883 (PMC3877100; doi:10.1371/journal.pone.0083883)
Supplement: Table S2 — PERMANOVA of anemone abundance based on Bray Curtis similarity measure and b) Pairwise testing for the interaction Ye. Data were dispersion weighted and square root transformed. Bold type denotes a significant result. (DOCX) [file pone.0083883.s002.docx]

Table S2: PERMANOVA of anemone abundance based on Bray Curtis similarity measure and b) Pairwise testing for the interaction Ye. Data were dispersion weighted and square root transformed. Bold type denotes a significant result.

| **a)** |  |  |  |  |  |
| --- | --- | --- | --- | --- | --- |
| **Source** | **df** | **SS** | **MS** | **F** | **P** |
| Year Ye | 3 | 18.13 | 6.0427 | 2.86 | **0.0411** |
| Treatment Tr | 3 | 5.22 | 1.7408 | 0.67 | 0.6119 |
| Area Ar (Tr) | 15 | 37.04 | 2.469 | 2.51 | **0.0097** |
| YexTr | 9 | 26.74 | 2.9709 | 1.49 | 0.1865 |
| Site(Ar(Tr)) | 50 | 44.53 | 0.8905 | 1.54 | **0.0403** |
| YexAr(Tr) | 45 | 77.05 | 1.7122 | 2.96 | **0.0001** |
| Residual | 110 | 63.66 | 0.57874 |  |  |
| Total | 235 | 272.36 |  |  |  |

| **b)** |  | |
| --- | --- | --- |
|  | **Ye** | |
| **Groups** | **t** | **P** |
| 2008, 2009 | 2.72 | **0.0159** |
| 2008, 2010 | 3.20 | **0.0057** |
| 2008, 2011 | 2.66 | **0.0177** |
| 2009, 2010 | 0.40 | 0.7011 |
| 2009, 2011 | 0.31 | 0.7636 |
| 2010, 2011 | 0.37 | 0.7092 |
