# Supplementary material for: Recovery of a Temperate Reef Assemblage in a Marine Protected Area following the Exclusion of Towed Demersal Fishing
Source: PLoS One. 2013 Dec 31;8(12):e83883. doi: 10.1371/journal.pone.0083883 (PMC3877100; doi:10.1371/journal.pone.0083883)
Supplement: Table S3 — PERMANOVA of Pentapora fascialis abundance based on Bray Curtis similarity measure and b) Pairwise testing for the interaction YexTr. Data were dispersion weighted and square root transformed. Bold type denotes a significant result. (DOCX) [file pone.0083883.s003.docx]

Table S3: PERMANOVA of *Pentapora fascialis* abundance based on Bray Curtis similarity measure and b) Pairwise testing for the interaction YexTr. Data were dispersion weighted and square root transformed. Bold type denotes a significant result.

| **a)** |  |  |  |  |  |
| --- | --- | --- | --- | --- | --- |
| **Source** | **df** | **SS** | **MS** | **F** | **P** |
| Year Ye | 3 | 4.90 | 1.6346 | 11.74 | **0.0001** |
| Treatment Tr | 3 | 15.86 | 5.2879 | 7.06 | **0.0031** |
| Area Ar (Tr) | 15 | 10.03 | 0.66853 | 3.67 | **0.0004** |
| YexTr | 9 | 4.24 | 0.47075 | 3.70 | **0.0013** |
| Site(Ar(Tr)) | 50 | 8.11 | 0.16212 | 2.29 | **0.0007** |
| YexAr(Tr) | 45 | 4.76 | 0.10583 | 1.49 | 0.0526 |
| Residual | 110 | 7.79 | 0.07 |  |  |
| Total | 235 | 55.69 |  |  |  |

| **b)** |  | |  | |  | |  | |
| --- | --- | --- | --- | --- | --- | --- | --- | --- |
|  | **2008** | | **2009** | | **2010** | | **2011** | |
| **Groups** | **t** | **P** | **t** | **P** | **t** | **P** | **t** | **P** |
| CC, NC | 1.79 | 0.0998 | 1.13 | 0.2947 | 1.75 | 0.097 | 3.73 | **0.0138** |
| CC, NOC | 3.09 | **0.0178** | 2.66 | **0.0298** | 1.09 | 0.3131 | 1.49 | 0.1898 |
| CC, FOC | 2.72 | **0.0242** | 2.58 | **0.0336** | 1.94 | 0.0774 | 5.74 | **0.0003** |
| NC, NOC | 2.33 | **0.0368** | 1.82 | 0.0932 | 2.56 | **0.0419** | 2.45 | **0.0274** |
| NC, FOC | 1.66 | 0.1237 | 1.73 | 0.1136 | 0.86 | 0.5573 | 1.17 | 0.2959 |
| NOC, FOC | 1.50 | 0.1722 | 0.69 | 0.5823 | 2.70 | **0.025** | 3.06 | **0.0204** |
