# Supplementary material for: Recovery of a Temperate Reef Assemblage in a Marine Protected Area following the Exclusion of Towed Demersal Fishing
Source: PLoS One. 2013 Dec 31;8(12):e83883. doi: 10.1371/journal.pone.0083883 (PMC3877100; doi:10.1371/journal.pone.0083883)
Supplement: Table S4 — PERMANOVA of Eunicella verrucosa abundance based on Bray Curtis similarity measure. Data were dispersion weighted and square root transformed. Bold type denotes a significant result. (DOCX) [file pone.0083883.s004.docx]

Table S4: PERMANOVA of *Eunicella verrucosa* abundance based on Bray Curtis similarity measure. Data were dispersion weighted and square root transformed. Bold type denotes a significant result.

| **Source** | ***df*** | **SS** | **MS** | ***F*** | **P** |
| --- | --- | --- | --- | --- | --- |
| Year Ye | 3 | 0.23 | 0.07616 | 1.80 | 0.158 |
| Treatment Tr | 3 | 3.28 | 1.093 | 1.07 | 0.39 |
| Area Ar (Tr) | 15 | 13.58 | 0.90555 | 17.99 | **0.0001** |
| YexTr | 9 | 0.53 | 0.059431 | 1.91 | 0.0624 |
| Site(Ar(Tr)) | 59 | 2.55 | 0.043174 | 1.79 | **0.0198** |
| YexAr(Tr) | 45 | 1.19 | 0.026405 | 1.09 | 0.3546 |
| Residual | 117 | 2.82 | 0.024136 |  |  |
| Total | 251 | 24.19 |  |  |  |
