# Supplementary material for: Recovery of a Temperate Reef Assemblage in a Marine Protected Area following the Exclusion of Towed Demersal Fishing
Source: PLoS One. 2013 Dec 31;8(12):e83883. doi: 10.1371/journal.pone.0083883 (PMC3877100; doi:10.1371/journal.pone.0083883)
Supplement: Table S5 — PERMANOVA of Phallusia mammillata abundance based on Bray Curtis similarity measure. Data were dispersion weighted and square root transformed. Bold type denotes a significant result. (DOCX) [file pone.0083883.s005.docx]

Table S5: PERMANOVA of *Phallusia mammillata* abundance based on Bray Curtis similarity measure. Data were dispersion weighted and square root transformed. Bold type denotes a significant result.

| **Source** | ***df*** | **SS** | **MS** | ***F*** | **P** |
| --- | --- | --- | --- | --- | --- |
| Year Ye | 3 | 0.14 | 0.045614 | 2.10 | 0.1118 |
| Treatment Tr | 3 | 1.79 | 0.59747 | 5.80 | **0.0053** |
| Area Ar (Tr) | 15 | 1.37 | 0.09112 | 2.95 | **0.0056** |
| YexTr | 9 | 0.38 | 0.042548 | 2.10 | 0.0532 |
| Site(Ar(Tr)) | 59 | 1.57 | 0.026693 | 3.62 | **0.0001** |
| YexAr(Tr) | 45 | 0.73 | 0.016187 | 2.20 | **0.0033** |
| Residual | 117 | 0.86 | 0.007366 |  |  |
| Total | 251 | 6.84 |  |  |  |
