# Supplementary material for: Recovery of a Temperate Reef Assemblage in a Marine Protected Area following the Exclusion of Towed Demersal Fishing
Source: PLoS One. 2013 Dec 31;8(12):e83883. doi: 10.1371/journal.pone.0083883 (PMC3877100; doi:10.1371/journal.pone.0083883)
Supplement: Table S6 — PERMANOVA of Cellepora pumicosa abundance based on Bray Curtis similarity measure. Data were dispersion weighted and square root transformed. Bold type denotes a significant result. (DOCX) [file pone.0083883.s006.docx]

Table S6: PERMANOVA of *Cellepora pumicosa* abundance based on Bray Curtis similarity measure. Data were dispersion weighted and square root transformed. Bold type denotes a significant result.

| **Source** | ***df*** | **SS** | **MS** | ***F*** | **P** |
| --- | --- | --- | --- | --- | --- |
| Year Ye | 3 | 16.20 | 5.399 | 2.09 | 0.1077 |
| Treatment Tr | 3 | 84.97 | 28.322 | 5.29 | **0.0072** |
| Area Ar (Tr) | 15 | 71.54 | 4.7693 | 7.85 | **0.0001** |
| YexTr | 9 | 20.33 | 2.2593 | 0.94 | 0.5019 |
| Site(Ar(Tr)) | 50 | 27.02 | 0.54045 | 1.09 | 0.3488 |
| YexAr(Tr) | 45 | 93.97 | 2.0882 | 4.20 | **0.0001** |
| Residual | 110 | 54.67 | 0.49697 |  |  |
| Total | 235 | 368.69 |  |  |  |
