# Supplementary material for: Recovery of a Temperate Reef Assemblage in a Marine Protected Area following the Exclusion of Towed Demersal Fishing
Source: PLoS One. 2013 Dec 31;8(12):e83883. doi: 10.1371/journal.pone.0083883 (PMC3877100; doi:10.1371/journal.pone.0083883)
Supplement: Table S7 — PERMANOVA of Chaetopterus variopedatus abundance based on Bray Curtis similarity measure and b) Pairwise testing for the interaction YexTr. Data were dispersion weighted and square root transformed. Bold type denotes a significant result. (DOCX) [file pone.0083883.s007.docx]

Table S7: PERMANOVA of *Chaetopterus variopedatus* abundance based on Bray Curtis similarity measure and b) Pairwise testing for the interaction YexTr. Data were dispersion weighted and square root transformed. Bold type denotes a significant result.

| **a)** |  |  |  |  |  |
| --- | --- | --- | --- | --- | --- |
| **Source** | ***df*** | **SS** | **MS** | ***F*** | **P** |
| Year Ye | 3 | 2.40 | 0.80059 | 1.66 | 0.1863 |
| Treatment Tr | 3 | 4.44 | 1.4803 | 1.18 | 0.351 |
| Area Ar (Tr) | 15 | 17.04 | 1.1363 | 4.41 | **0.0003** |
| YexTr | 9 | 8.80 | 0.97741 | 2.08 | **0.0484** |
| Site(Ar(Tr)) | 50 | 11.59 | 0.23184 | 1.10 | 0.3493 |
| YexAr(Tr) | 45 | 18.19 | 0.40417 | 1.92 | **0.0032** |
| Residual | 110 | 23.17 | 0.21062 |  |  |
| Total | 235 | 85.63 |  |  |  |

| **b)** |  | |  | |  | |  | |
| --- | --- | --- | --- | --- | --- | --- | --- | --- |
|  | **2008** | | **2009** | | **2010** | | **2011** | |
| **Groups** | **t** | **P** | **t** | **P** | **t** | **P** | **t** | **P** |
| CC, NC | 0.30 | 0.9793 | 0.88 | 0.4381 | 1.33 | 0.2409 | 1.83 | 0.0976 |
| CC, NOC | 0.36 | 0.9153 | 1.16 | 0.2967 | 0.43 | 0.839 | 1.00 | 1 |
| CC, FOC | 2.05 | 0.0706 | 1.46 | 0.1785 | 0.55 | 0.7099 | 0.26 | 0.8439 |
| NC, NOC | 0.45 | 0.8837 | 0.56 | 0.7674 | 2.38 | **0.0368** | 3.12 | **0.0127** |
| NC, FOC | 2.43 | **0.0327** | 1.02 | 0.3455 | 1.39 | 0.2066 | 1.94 | 0.0794 |
| NOC, FOC | 2.20 | 0.0574 | 0.79 | 0.5391 | 0.39 | 0.8914 | 1.84 | 0.1027 |
