# Supplementary material for: Recovery of a Temperate Reef Assemblage in a Marine Protected Area following the Exclusion of Towed Demersal Fishing
Source: PLoS One. 2013 Dec 31;8(12):e83883. doi: 10.1371/journal.pone.0083883 (PMC3877100; doi:10.1371/journal.pone.0083883)
Supplement: Table S8 — PERMANOVA of hydroid abundance based on Bray Curtis similarity measure and b) Pairwise testing for the interactions Tr and Ye. Data were dispersion weighted and square root transformed. Bold type denotes a significant result. (DOCX) [file pone.0083883.s008.docx]

Table S8: PERMANOVA of hydroid abundance based on Bray Curtis similarity measure and b) Pairwise testing for the interactions Tr and Ye. Data were dispersion weighted and square root transformed. Bold type denotes a significant result.

| **a)** |  |  |  |  |  |
| --- | --- | --- | --- | --- | --- |
| **Source** | ***df*** | **SS** | **MS** | ***F*** | **P** |
| Year Ye | 3 | 33.22 | 11.075 | 5.07 | **0.0032** |
| Treatment Tr | 3 | 51.86 | 17.287 | 4.54 | **0.0177** |
| Area Ar (Tr) | 15 | 51.21 | 3.4136 | 2.79 | **0.0041** |
| YexTr | 9 | 15.82 | 1.7581 | 0.89 | 0.5478 |
| Site(Ar(Tr)) | 50 | 54.93 | 1.0985 | 1.99 | **0.0028** |
| YexAr(Tr) | 45 | 78.36 | 1.7414 | 3.15 | **0.0001** |
| Residual | 110 | 60.81 | 0.55282 |  |  |
| Total | 235 | 346.21 |  |  |  |

| **b)** |  | |  |  |  | |
| --- | --- | --- | --- | --- | --- | --- |
|  | **Tr** | |  |  | **Ye** | |
| **Groups** | **t** | **P** |  | **Groups** | **T** | **P** |
| CC, NC | 1.56 | 0.1519 |  | 2008, 2009 | 1.36 | 0.1943 |
| CC, NOC | 3.44 | **0.0117** |  | 2008, 2010 | 2.14 | **0.0458** |
| CC, FOC | 3.22 | **0.0117** |  | 2008, 2011 | 3.89 | **0.0006** |
| NC, NOC | 0.79 | 0.4893 |  | 2009, 2010 | 1.07 | 0.2977 |
| NC, FOC | 2.16 | 0.0576 |  | 2009, 2011 | 2.34 | **0.0266** |
| NOC, FOC | 1.61 | 0.1411 |  | 2010, 2011 | 1.50 | 0.147 |
