# Supplementary material for: Recovery of a Temperate Reef Assemblage in a Marine Protected Area following the Exclusion of Towed Demersal Fishing
Source: PLoS One. 2013 Dec 31;8(12):e83883. doi: 10.1371/journal.pone.0083883 (PMC3877100; doi:10.1371/journal.pone.0083883)
Supplement: Table S9 — PERMANOVA of branching sponge abundance based on Bray Curtis similarity measure and b) Pairwise testing for the interaction YexTr. Data were dispersion weighted and square root transformed. Bold type denotes a significant result. (DOCX) [file pone.0083883.s009.docx]

Table S9: PERMANOVA of branching sponge abundance based on Bray Curtis similarity measure and b) Pairwise testing for the interaction YexTr. Data were dispersion weighted and square root transformed. Bold type denotes a significant result.

| **a)** |  |  |  |  |  |
| --- | --- | --- | --- | --- | --- |
| **Source** | ***df*** | **SS** | **MS** | ***F*** | **P** |
| Year Ye | 3 | 0.82 | 0.27231 | 6.50 | **0.0012** |
| Treatment Tr | 3 | 1.44 | 0.48044 | 7.11 | **0.0023** |
| Area Ar (Tr) | 15 | 0.89 | 0.05938 | 4.22 | **0.0002** |
| YexTr | 9 | 0.66 | 0.073507 | 1.92 | 0.0724 |
| Site(Ar(Tr)) | 59 | 0.74 | 0.012463 | 0.93 | 0.5785 |
| YexAr(Tr) | 45 | 1.44 | 0.031911 | 2.38 | **0.0006** |
| Residual | 117 | 1.57 | 0.013399 |  |  |
| Total | 251 | 7.55 |  |  |  |

| **b)** |  | |  | |  | |  | |
| --- | --- | --- | --- | --- | --- | --- | --- | --- |
|  | **2008** | | **2009** | | **2010** | | **2011** | |
| **Groups** | **t** | **P** | **T** | **P** | **t** | **P** | **T** | **P** |
| CC, NC | 0.42 | 0.9266 | 0.31 | 0.9897 | 0.27 | 0.9979 | 2.31 | **0.0427** |
| CC, NOC | 7.58 | **0.0005** | 2.23 | 0.0606 | 2.03 | 0.0818 | 3.12 | **0.0274** |
| CC, FOC | 2.25 | 0.0525 | 2.23 | 0.0522 | 1.99 | 0.0789 | 3.64 | **0.0089** |
| NC, NOC | 1.43 | 0.186 | 2.76 | **0.0154** | 2.34 | **0.034** | 2.02 | **0.043** |
| NC, FOC | 0.85 | 0.4598 | 2.80 | **0.0137** | 2.31 | **0.0377** | 2.47 | **0.0112** |
| NOC, FOC | 1.15 | 0.3301 | 0.30 | 0.9519 | 0.77 | 0.531 | 0.80 | 0.4764 |
