# Supplementary material for: Recovery of a Temperate Reef Assemblage in a Marine Protected Area following the Exclusion of Towed Demersal Fishing
Source: PLoS One. 2013 Dec 31;8(12):e83883. doi: 10.1371/journal.pone.0083883 (PMC3877100; doi:10.1371/journal.pone.0083883)
Supplement: Table S10 — PERMANOVA of Pecten maximus abundance based on Bray Curtis similarity measure and b) Pairwise testing for the interaction YexTr. Data were dispersion weighted and square root transformed. Bold type denotes a significant result. (DOCX) [file pone.0083883.s010.docx]

Table S10: PERMANOVA of *Pecten maximus* abundance based on Bray Curtis similarity measure and b) Pairwise testing for the interaction YexTr. Data were dispersion weighted and square root transformed. Bold type denotes a significant result.

| **a)** |  |  |  |  |  |
| --- | --- | --- | --- | --- | --- |
| **Source** | ***df*** | **SS** | **MS** | ***F*** | **P** |
| Year Ye | 3 | 0.73 | 0.24209 | 9.44 | **0.0002** |
| Treatment Tr | 3 | 1.03 | 0.34424 | 2.81 | 0.0672 |
| Area Ar (Tr) | 15 | 1.63 | 0.10888 | 3.34 | **0.0008** |
| YexTr | 9 | 0.69 | 0.076124 | 3.33 | **0.0035** |
| Site(Ar(Tr)) | 59 | 1.68 | 0.028537 | 1.77 | **0.0186** |
| YexAr(Tr) | 45 | 0.83 | 0.018514 | 1.15 | 0.2887 |
| Residual | 117 | 1.89 | 0.016139 |  |  |
| Total | 251 | 8.48 |  |  |  |

| **b)** |  | |  | |  | |  | |
| --- | --- | --- | --- | --- | --- | --- | --- | --- |
|  | **2008** | | **2009** | | **2010** | | **2011** | |
| **Groups** | **t** | **P** | **T** | **P** | **t** | **P** | **t** | **P** |
| CC, NC | 0.71 | 0.6109 | 1.11 | 0.3077 | 1.71 | 0.1005 | 1.28 | 0.2774 |
| CC, NOC | 0.62 | 0.6657 | 0.39 | 0.8804 | 0.63 | 0.6443 | 1.54 | 0.1995 |
| CC, FOC | 0.41 | 0.8597 | 0.94 | 0.4049 | 1.59 | 0.1533 | 2.28 | 0.0814 |
| NC, NOC | 0.73 | 0.6119 | 1.23 | 0.2435 | 2.36 | **0.0344** | 2.13 | 0.082 |
| NC, FOC | 0.80 | 0.4931 | 0.65 | 0.6193 | 3.27 | **0.0068** | 2.66 | **0.0195** |
| NOC, FOC | 0.71 | 0.5767 | 1.13 | 0.3096 | 1.34 | 0.2203 | 0.34 | 0.7319 |
