# Supplementary material for: Recovery of a Temperate Reef Assemblage in a Marine Protected Area following the Exclusion of Towed Demersal Fishing
Source: PLoS One. 2013 Dec 31;8(12):e83883. doi: 10.1371/journal.pone.0083883 (PMC3877100; doi:10.1371/journal.pone.0083883)
Supplement: Table S11 — PERMANOVA of Necora puber abundance based on Bray Curtis similarity measure and b) Pairwise testing for the interaction Ye. Data were dispersion weighted and square root transformed. Bold type denotes a significant result. (DOCX) [file pone.0083883.s011.docx]

Table S11: PERMANOVA of *Necora puber* abundance based on Bray Curtis similarity measure and b) Pairwise testing for the interaction Ye. Data were dispersion weighted and square root transformed. Bold type denotes a significant result.

| **a)** |  |  |  |  |  |
| --- | --- | --- | --- | --- | --- |
| **Source** | ***df*** | **SS** | **MS** | ***F*** | **P** |
| Year Ye | 3 | 0.02 | 0.006033 | 4.80 | **0.0039** |
| Treatment Tr | 3 | 0.02 | 0.006156 | 2.47 | 0.0955 |
| Area Ar (Tr) | 15 | 0.03 | 0.002242 | 1.74 | 0.0754 |
| YexTr | 9 | 0.01 | 0.001274 | 1.18 | 0.3189 |
| Site(Ar(Tr)) | 59 | 0.07 | 0.001155 | 1.75 | **0.0306** |
| YexAr(Tr) | 45 | 0.04 | 0.000956 | 1.45 | 0.085 |
| Residual | 117 | 0.08 | 0.000662 |  |  |
| Total | 251 | 0.27 |  |  |  |

| **b)** |  | |
| --- | --- | --- |
|  | **Ye** | |
| **Groups** | **t** | **P** |
| 2008, 2009 | 3.18 | **0.0052** |
| 2008, 2010 | 3.36 | **0.0039** |
| 2008, 2011 | 2.52 | **0.0175** |
| 2009, 2010 | 1.83 | 0.0848 |
| 2009, 2011 | 0.84 | 0.4037 |
| 2010, 2011 | 0.86 | 0.4021 |
