# Supplementary material for: Recovery of a Temperate Reef Assemblage in a Marine Protected Area following the Exclusion of Towed Demersal Fishing
Source: PLoS One. 2013 Dec 31;8(12):e83883. doi: 10.1371/journal.pone.0083883 (PMC3877100; doi:10.1371/journal.pone.0083883)
Supplement: Table S12 — PERMANOVA of Asterias rubens abundance based on Bray Curtis similarity measure. Data were dispersion weighted and square root transformed. Bold type denotes a significant result. (DOCX) [file pone.0083883.s012.docx]

Table S12: PERMANOVA of *Asterias rubens* abundance based on Bray Curtis similarity measure. Data were dispersion weighted and square root transformed. Bold type denotes a significant result.

| **Source** | ***df*** | **SS** | **MS** | ***F*** | **P** |
| --- | --- | --- | --- | --- | --- |
| Year Ye | 3 | 0.62 | 0.208 | 1.17 | 0.332 |
| Treatment Tr | 3 | 0.97 | 0.32181 | 0.33 | 0.8622 |
| Area Ar (Tr) | 15 | 13.14 | 0.87612 | 10.63 | **0.0001** |
| YexTr | 9 | 1.10 | 0.12225 | 0.78 | 0.6288 |
| Site(Ar(Tr)) | 59 | 4.19 | 0.071033 | 1.39 | 0.102 |
| YexAr(Tr) | 45 | 6.23 | 0.13835 | 2.70 | **0.0001** |
| Residual | 117 | 5.99 | 0.051171 |  |  |
| Total | 251 | 32.24 |  |  |  |
