# Supplementary material for: Recovery of a Temperate Reef Assemblage in a Marine Protected Area following the Exclusion of Towed Demersal Fishing
Source: PLoS One. 2013 Dec 31;8(12):e83883. doi: 10.1371/journal.pone.0083883 (PMC3877100; doi:10.1371/journal.pone.0083883)
Supplement: Table S13 — PERMANOVA of Cancer pagurus abundance based on Bray Curtis similarity measure and b) Pairwise testing for the interaction Tr. Data were dispersion weighted and square root transformed. Bold type denotes a significant result. (DOCX) [file pone.0083883.s013.docx]

Table S13: PERMANOVA of *Cancer pagurus* abundance based on Bray Curtis similarity measure and b) Pairwise testing for the interaction Tr. Data were dispersion weighted and square root transformed. Bold type denotes a significant result.

| **a)** |  |  |  |  |  |
| --- | --- | --- | --- | --- | --- |
| **Source** | ***df*** | **SS** | **MS** | ***F*** | **P** |
| Year Ye | 3 | 0.00 | 0.000138 | 0.78 | 0.5009 |
| Treatment Tr | 3 | 0.00 | 0.001066 | 3.08 | **0.0473** |
| Area Ar (Tr) | 15 | 0.00 | 0.000313 | 0.99 | 0.4584 |
| YexTr | 9 | 0.00 | 0.000129 | 0.73 | 0.6773 |
| Site(Ar(Tr)) | 59 | 0.02 | 0.000308 | 1.20 | 0.2184 |
| YexAr(Tr) | 45 | 0.01 | 0.000214 | 0.84 | 0.7326 |
| Residual | 117 | 0.03 | 0.000256 |  |  |
| Total | 251 | 0.07 |  |  |  |

| **b)** |  | |
| --- | --- | --- |
|  | **Tr** | |
| **Groups** | **t** | **P** |
| CC, NC | 1.82 | 0.0942 |
| CC, NOC | 0.38 | 0.8589 |
| CC, FOC | 2.21 | 0.0557 |
| NC, NOC | 1.98 | 0.0762 |
| NC, FOC | 1.44 | 0.176 |
| NOC, FOC | 2.32 | **0.0489** |
